# Association between perceived life stress and subjective well-being among Chinese perimenopausal women: a moderated mediation analysis

**DOI:** 10.7717/peerj.12787

**Published:** 2022-01-18

**Authors:** Xiangrong Li, Zheng Ren, Tianliang Ji, Hong Shi, Hanfang Zhao, Minfu He, Xinwen Fan, Xia Guo, Shuang Zha, Shuyin Qiao, Yuyu Li, Yajiao Pu, Hongjian Liu, Xiumin Zhang

**Affiliations:** 1 Department of Social Medicine and Health Management, School of Public Health, Jilin University, Changchun, China; 2 Department of Cardiovascular Medicine, The First Hospital of Jilin University, Changchun, China; 3 Department of Epidemiology and Biostatistics, School of Public Health, Jilin University, Changchun, China

**Keywords:** Perceived life stress, Depressive symptoms, Interests/hobbies, Subjective well-being, Perimenopausal women

## Abstract

**Background:**

The impact of perceived life stress on subjective well-being has been well-established; while few studies have explored the mediating and moderating mechanisms of the association between perceived life stress and subjective well-being among perimenopausal women. This study is aimed at exploring the mediating effect of depressive symptoms and the role of interests/hobbies as a moderator in the association between perceived life stress and subjective well-being among perimenopausal women.

**Methods:**

The participants were 1,104 perimenopausal women at the age of 40 to 60, who were asked to complete a paper-based questionnaire. A single item was used to measure self-perceived life stress and interests/hobbies. The Zung Self-rating Depression Scale (SDS) and Subjective Well-being Scale for Chinese Citizens (SWBS-CC) were applied to assess both depressive symptoms and subjective well-being. Multiple linear regression analysis and the PROCESS macro were adopted to analyse not only the mediating effect of depressive symptoms but also the moderating role of interests/hobbies.

**Results:**

Perceived life stress was negatively associated with subjective well-being (*B* =  − 1.424, *β* =  − 0.101, *P* < 0.001). The impact of perceived life stress on subjective well-being was partially mediated by depressive symptoms (mediation effect = −0.760, 95% confidence intervals (CI) [−1.129, −0.415]). In addition, the interaction term between depressive symptoms and interests/hobbies was significantly related to subjective well-being (*β* =  − 0.060, *P* < 0.05), indicating moderating effect. Moderated mediation had a significant index (Index = −0.220, SE = 0.099, 95% CI [−0.460, −0.060]).

**Conclusions:**

Perceived life stress was negatively related to subjective well-being. The impact of perceived life stress on subjective well-being was mediated by depressive symptoms. Besides, interests/hobbies moderated the indirect effect of depressive symptoms on the relationship between perceived life stress and subjective well-being.

## Introduction

Also called “the menopausal transition”, perimenopause is a midlife transition period experienced by women and characterized by declining ovarian function and subsequent anovulatory cycles, usually beginning in the mid-to-late 40s (McNamara, Batur & DeSapri, 2015; Grady, 2006). Owing to the reductions or fluctuations in sex hormones resulting from decreased ovarian function, some women often face a variety of physical and psychological symptoms clinically known as “perimenopausal syndrome” and featuring insomnia, tiredness, hot flashes, mood swings, *etc.* (Meng et al., 2017). A large number of studies have found that most symptoms have a negative impact on the quality of life (Greenblum et al., 2013; Ngai, 2019). Women with more perimenopausal symptoms report worse quality of life that will be impaired as perimenopausal symptoms augment (Larroy et al., 2020). Subjective well-being is one of the indicators to measure the quality of life, which is defined as people’s assessments of their lives and includes the emotional responses of individuals, domain satisfactions as well as the overall judgments of life satisfaction (Diener & Chan, 2011; Diener et al., 1999). With the acceleration of population aging and the extension of life expectancy, it has become important to improve and pay attention to the subjective well-being of perimenopausal women.

Life stress has a complicated construct covering a variety of adverse social-environmental experiences called stressors which can vary by frequency, severity, timing and duration (Slavich, 2019; Slavich, 2016). During this transition, women can face a number of life stressors, including but not limited to aging, caring for parents, illness of individuals or families, career or marriage shifts and body changes, which could exert a negative impact on their physical or psychological health and subjective well-being. At an individual level, stress reflects a negative affective state and shows a strong correlation with affective experience (Ng et al., 2009) and negative well-being (Martin, Grunendahl & Martin, 2001). From a pathological point of view, a growing body of evidence has suggested that perceived stress is associated with cardiovascular disease (Katsarou, Triposkiadis & Panagiotakos, 2013; Albert et al., 2017), atrial fibrillation (O’Neal et al., 2015), and hypertension (Wiernik et al., 2014; Spruill et al., 2019). A study on urban women in African America found that higher perceived stress is associated with lower health status and well-being (Young et al., 2004). Hence, it is reasonable to postulate that a high level of perceived life stress is significantly associated with a low level of subjective well-being. On this basis, a recent study verified the relationship between stress and global subjective well-being, indicating that stress is related to all three major forms of subjective well-being, including low negative feelings, positive feelings, and life satisfaction (Ng & Diener, 2021). Based on the above analysis, we put forward the first hypothesis:

Hypothesis 1. Perceived life stress was correlated with the subjective well-being of perimenopausal women.

The hypothesized association between perceived life stress and subjective well-being is likely to be explained partially by depressive symptoms. Stress plays an important role in the development, continuation, and exacerbation of mood disorders in the lives of women (Alexander et al., 2007). Perimenopause refers to a period when women are more prone to depressive symptoms along with higher severity of symptoms compared with premenopause (De Kruif, Spijker & Molendijk, 2016). The prevalence of depressive symptoms in Chinese perimenopausal women has been reported to range from 25.99% to 68.70% (Li et al., 2016; Yan et al., 2018; Hu, Tang & Zhang, 2020). Stress plays an important role in the generation and development of depressive symptoms. A strong association of depressive symptoms with stress provides many opportunities to understand the vulnerabilities and mechanisms affecting depressive symptoms. Stress can trigger certain diatheses and consequently causes depression (Monroe & Simons, 1991). In addition, a stress exposure model of depression implies that stress increases individual susceptibility to depression (Liu & Alloy, 2010). Stress leading to brain disorders is believed to underlie specific components of depressive syndromes (Van Praag, 2004). Biologically, sustained stress causes the change of 5-hydroxytryptamine and cortisol systems, which is similar to that observed in a subtype of depression (Van Praag, 2004). A nationwide study of middle-aged menopausal women in Korea noticed that a high level of perceived stress is a factor to influence depressive symptoms (Kim, 2020). Depressive symptoms are associated with subjective well-being. Given that depressive symptoms play a role as a disorder of emotional dysregulation and sustained negative affect, a large number of studies have found that depressive symptoms exert a negative impact on subjective well-being. In other words, the higher the level of depressive symptoms was, the lower the level of subjective well-being was (Malone & Wachholtz, 2018; Sun, Bao & Wei, 2016). Perceived stress may increase the incidence of depressive symptoms, which in turn could have an important impact on subjective well-being. Considering these interrelationships, it is plausible that the relationship between perceived life stress and subjective well-being may be mediated by depressive symptoms. Taking the above reasoning and evidence together, we propose the second hypothesis:

Hypothesis 2. Depressive symptoms mediate the relationship between perceived life stress and subjective well-being.

Based on the broaden-and-build theory of positive emotions, interests as a positive emotion can broaden people’s repertoires of short-lived thought actions to build personal resources in a range from intellectual and physical ones to psychological and social ones (Fredrickson, 2001). This means that people should cultivate positive emotions as a way of achieving psychological growth and improving subjective well-being over time. Interests spark the urge to explore (Fredrickson, 2004), and empirical studies have found that interests/hobbies are beneficial for alleviating depressive symptoms (Li, Bo & Lv, 2015; Wong et al., 2014) and promoting subjective well-being (Fang & Jin, 2021). The broaden-and-build model of positive emotions holds that positive emotions may correct or eliminate the consequences of negative emotions, which is called undo hypothesis (Fredrickson, 1998). Previous research has also proposed physiological markers of broadening effects, holding that heart rate variability underlies positive emotions and the ability to minimize negative reactions (McCraty et al., 1995). Heart rate variability is an effective tool to measure and regulate emotional responses (Zhu, Ji & Liu, 2019), and the major supporting theories contain the model of neurovisceral integration and the polyvagal theory (Appelhans & Luecken, 2006). Positive emotions can buffer against the negative physical and mental consequences of stress by promoting friendship, enhancing psychological resilience, and improving coping strategies (Garland, Gaylord & Fredrickson, 2011). According to the theory, interests/hobbies as a kind of positive emotion can play a buffering role in the association between perceived life stress/depressive symptoms and subjective well-being.

The dynamic model of affect (Zautra et al., 2001) holds that positive and negative emotions are relatively independent in general situations, while an inverse correlation between positive and negative emotions increases dramatically in the event of stress. Positive emotions reduce the size of the relationship between negative emotions and stress. In addition, a previous study found that the promotion of positive emotions after stress predicts a decrease in concurrent negative emotions (Monfort, Stroup & Waugh, 2015). Therefore, it can be assumed that interests/hobbies as a positive emotion may have a buffering role in the association between perceived life stress and depressive symptoms. Based on the theoretical and empirical literature above, we propose the third hypothesis:

Hypothesis 3. Interests/hobbies can moderate the direct and indirect association between perceived life stress and subjective well-being *via* depressive symptoms.

**Figure 1:**
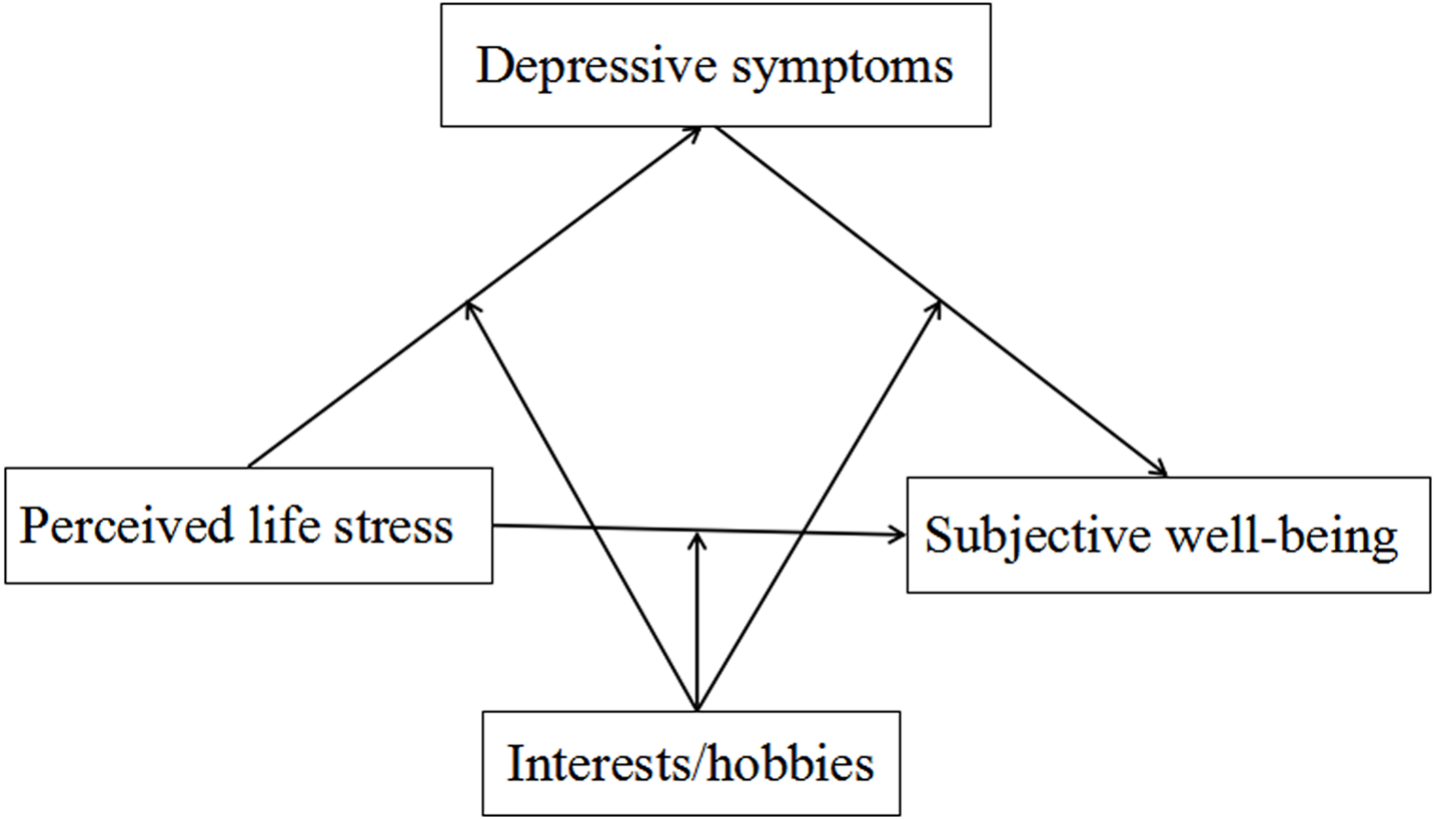
The conceptual framework of the moderated mediation model.

It is significant to clarify the mediating and moderating mechanisms underlying the relationship between the perceived life stress and the subjective well-being of perimenopausal women. The aims of this study are as follows: (1) investigate the association of perceived life stress with subjective well-being; (2) examine whether depressive symptoms can mediate the relationship between perceived life stress and subjective well-being; (3) clarify whether interests/hobbies moderate the direct and indirect association between perceived life stress and subjective well-being through depressive symptoms. Figure 1 illustrates the proposed model.

## Materials & Methods

### Study participants

Performed in the Medical Examination Center of the First Hospital of Jilin University in northeastern China from July to September in 2019, this survey invited a total of 1,144 perimenopausal women aged 40–60 and have participated in a regular physical examination to complete a paper-based and self-administered questionnaire. In this study, exclusion criteria were any of the following self-reports: (1) Incomplete data and (2) cancer diseases. A total of 1,104 survey responses were valid. This survey obtained informed consent from all the participants enrolled in the study which received approval from the Ethics Committee of Jilin University School of Public Health (Reference Number: 2019-07-07).

### Basic information

Basic information included age, marital status, employment status, educational level, average monthly personal income, self-rated good family relationship, self-rated health, smoking status, drinking status, menstrual status, the use of progesterone/estrogen therapy, sleep quality and anxiety symptoms. Age was divided into four categories, namely 40–44, 45–50, 51–55 and 56–60. Marital status consisted of married and unmarried. Employment status was classified as employed and unemployed. Educational level was composed of three classes, including high school and below, junior college, bachelor degree and above. Average monthly personal income was made up of ≤3,999, 4,000–7,999, and ≥8,000 RMB. Self-rated good family relationship was evaluated by asking participants how they rate their family relationship, with response options of very good, good, fair, poor and very poor, and categorized into yes (very good/good) and no (fair/poor/very poor). Self-rated health was examined by the question “How do you rate your health?” with answers on a five-point scale: very good, good, fair, poor, and very poor, followed by division into good (good/very good), fair, and poor (poor/very poor). According to the current smoking or drinking situation, smoking status was categorized into two groups: yes (current smoking) and no (past and never smoking), and drinking status was divided into two groups: yes (current drinking) and no (past and never drinking). Menstrual status was composed of regular menstruation, irregular menstruation and menopause. The use of progesterone/estrogen therapy was measured by the question “Have you used estrogen/progestogen therapy?” with answers classified into two groups: no and yes. The Pittsburgh Sleep Quality Index (PSQI) was adopted to measure sleep quality (Buysse et al., 1989), including subjective sleep quality, sleep efficiency, sleep duration, sleep latency, sleep disturbance, sleep medication use and daytime dysfunction. The total PSQI score is the sum of seven component scores with a range of 0-21, and higher scores indicate poorer sleep quality. Sleep quality was categorized into two groups: good sleep quality was represented by PSQI score ≤ 7 and poor sleep quality was reflected by PSQI score > 7. Liu et al. (1996) prepared the Chinese version of PSQI, confirming that this scale has good internal consistency, retest reliability and validity. In this study, Cronbach’s α of PSQI was 0.834. Anxiety symptoms were measured using the Zung’s Self-rating Anxiety Scale (SAS) (Zung, 1971), which contains 20 items: five are reversely scored, and 15 are positively scored. The final score was set at between 25 and 100. The presence of anxiety symptoms was indicated when the SAS score ≥ 50. Cronbach’s α of SAS was 0.771 in this study.

### Measures

#### Perceived life stress

The independent variable was the perceived life stress of the survey participants evaluated by the question “How do you rate your current life stress?” The answers of the participants were included in a five-point scale, which were the very low, low, fair, high and very high (marked as 1, 2, 3, 4, and 5, respectively). Perceived life stress would be higher when the score was higher.

#### Depressive symptoms

Depressive symptoms were evaluated by the Zung Self-Rating Depression Scale (SDS) (Zung, 1965), which consists of 20 items and scores from 1 to 4 on a four-point rating scale varying from none or occasionally to most or always. The total raw score ranges between 20 and 80. The level of depressive symptoms would be higher when the score was higher. The reliability and validity of the SDS have been supported in samples of Chinese rural women (Peng et al., 2013). In this study, the Cronbach’s α for SDS was 0.858.

#### Interests/hobbies

Interests/hobbies were assessed by the question “How do you rate your interests/hobbies?”. The choice responses of the participants were on a 5-point scale, including the very extensive, extensive, fair, not extensive and very not extensive (marked as 1, 2, 3, 4 and 5, respectively). According to the extensive range of interests/hobbies, they were divided into two groups: yes (very extensive/extensive) and no (fair/not extensive/very not extensive).

### Subjective well-being

Developed by Xing (2003), Subjective Well-being Scale for Chinese Citizens (SWBS-CC) was adopted in order to evaluate the subjective well-being of samples whose scale consisted of nine positively scored items and 11 reverse ones and which was assessed on a six-point scale ranging between 1 (strongly disagree) and 6 (strongly agree). The total score varied between 20 and 120. The level of subjective well-being was higher when the score was higher. The Cronbach’s α of SWBS-CC was 0.871 in this study.

### Data analysis

Number (proportions), means and standard deviations (SDs) were adopted to describe categorical and continuous variables, respectively. Kolmogorov–Smirnov test was used to analyze the normal distribution of the perceived life stress, depressive symptoms, and subjective well-being. The absolute value of skewness and kurtosis coefficients was less than 2, which can approximately obey a normal distribution (Mardia & Foster, 1983). The Chi-square test and Fisher’s exact test were performed to compare the differences in basic information according to the classification of perceived life stress. The relationship between perceived life stress, depressive symptoms, and subjective well-being was examined using Pearson’s correlation analysis. Multiple linear regression analysis was adopted to both determine whether depressive symptoms mediated the link between perceived life stress and subjective well-being and explore whether interests/hobbies moderated the association between perceived life stress and subjective well-being, thereby controlling for basic information. After that, the PROCESS macro method was employed to reconfirm the results of the moderated mediation model. The confounding factors in each model included age, marital status, employment status, educational level, average monthly personal income, self-rated health, smoking status, drinking status, self-rated good family relationship, menstrual status, the use of progesterone/estrogen therapy, sleep quality, and anxiety symptoms. Statistical analyses were performed by using Statistical Product and Service Solutions (SPSS) 24.0 (IBM, Armonk, New York, the United States) and PROCESS version 2.16.

## Results

### Basic information and perceived life stress

The samples had an average age of 49.18, most of which were married (91.9%) and employed (78.6%). Menstrual status was divided into three groups: regular menstruation (38.2%), irregular menstruation (23.2%), and menopause (38.6%). It was reported that 56.7% of the samples completed a bachelor’s degree and above, and 21.4% reported their average monthly personal income greater than or equal to 8,000 RMB. A total of 901 participants (81.6%) reported good family relationships, and 5.6% reported the use of progesterone/estrogen therapy. In general, it is seen that 28.6% of the respondents had poor sleep quality, and 16.2% had anxiety symptoms. Table 1 displays the basic information of samples based on the categorized perceived life stress. There were statistical differences observed in the distribution of age, marital status, employment status, educational level, self-rated health, self-rated good family relationship, menstrual status, sleep quality, and anxiety symptoms among different perceived life stress groups.

**Table 1:** Basic information according to the categorized perceived life stress.

**Variable**		**Sample**	**Very low**	**Low**	**Fair**	**High**	**Very high**	***P***
**Age(years)**	40–44	223(20.2)	12(8.6)	40(14.7)	113(20.9)	53(40.2)	5(26.3)	**<0.001**
	45–50	468(42.4)	52(37.1)	128(47.1)	230(42.5)	52(39.4)	6(31.6)	
	51–55	237(21.5)	36(25.7)	52(19.1)	127(23.5)	17(12.9)	5(26.3)	
	56–60	176(15.9)	40(28.6)	52(19.1)	71(13.1)	10(7.6)	3(15.8)	
**Marital status**	Unmarried	89(8.1)	5(3.6)	25(9.2)	40(7.4)	15(11.4)	4(21.1)	0.028
	Married	1015(91.9)	135(96.4)	247(90.8)	501(92.6)	117(88.6)	15(78.9)	
**Employment status**	Unemployed	236(21.4)	52(37.1)	57(21.0)	104(19.2)	16(12.1)	7(36.8)	**<0.001**
	Employed	868(78.6)	88(62.9)	215(79.0)	437(80.8)	116(87.9)	12(63.2)	
**Educational level**	High school and below	251(22.7)	40(28.6)	45(16.5)	127(23.5)	31(23.5)	8(42.1)	0.025
	Junior college	227(20.6)	27(19.3)	60(22.1)	119(22.0)	20(15.1)	1(5.3)	
	Bachelor degree and above	626(56.7)	73(52.1)	167(61.4)	295(54.5)	81(61.4)	10(52.6)	
**Average monthly personal income(RMB)**	≤3999	329(29.8)	49(35.0)	63(23.2)	170(31.4)	40(30.3)	7(36.8)	0.115
	4000–7999	539(48.8)	67(47.9)	147(54.0)	254(47.0)	60(45.5)	11(57.9)	
	≥8000	236(21.4)	24(17.1)	62(22.8)	117(21.6)	32(24.2)	1(5.3)	
**Self-rated health**	Poor	107(9.7)	8(5.7)	18(6.6)	44(8.1)	30(22.7)	7(36.8)	**<0.001**
	Fair	556(50.4)	52(37.1)	106(39.0)	321(59.3)	69(52.3)	8(42.1)	
	Good	441(39.9)	80(57.1)	148(54.4)	176(32.5)	33(25.0)	4(21.1)	
Smoking status*	No	1079(97.7)	137(97.9)	268(98.5)	530(98.0)	127(96.2)	17(89.5)	0.110
	Yes	25(2.3)	3(2.1)	4(1.5)	11(2.0)	5(3.8)	2(10.5)	
**Drinking status**	No	905(82.0)	117(83.6)	223(82.0)	444(82.1)	103(78.0)	18(94.7)	0.444
	Yes	199(18.0)	23(16.4)	49(18.0)	97(17.9)	29(22.0)	1(5.3)	
**Self-rated good family relationship**	No	203(18.4)	13(9.3)	19(7.0)	117(21.6)	44(33.3)	10(52.6)	**<0.001**
	Yes	901(81.6)	127(90.7)	253(93.0)	424(78.4)	88(66.7)	9(47.4)	
**Menstrual status**	Regular menstruation	422(38.2)	37(26.4)	102(37.5)	208(38.4)	66(50.0)	9(47.4)	**<0.001**
	Irregular menstruation	256(23.2)	24(17.1)	61(22.4)	133(24.6)	36(27.3)	2(10.5)	
	Menopause	426(38.6)	79(56.4)	109(40.1)	200(37.0)	30(22.7)	8(42.1)	
**The use of progesterone/ estrogen therapy**	No	1042(94.4)	133(95.0)	252(92.6)	515(95.2)	123(93.2)	19(100.0)	0.433
	Yes	62(5.6)	7(5.0)	20(7.4)	26(4.8)	9(6.8)	0(0.0)	
**Sleep quality**	Good	788(71.4)	114(81.4)	211(77.6)	385(71.2)	70(53.0)	8(42.1)	**<0.001**
	Poor	316(28.6)	26(18.6)	61(22.4)	156(28.8)	62(47.0)	11(57.9)	
**Anxiety symptoms**	No	925(83.8)	128(91.4)	253(93.0)	446(82.4)	89(67.4)	9(47.4)	**<0.001**
	Yes	179(16.2)	12(8.6)	19(7.0)	95(17.6)	43(32.6)	10(52.6)	
**Total**		1104(100.0)	140(12.7)	272(24.6)	541(49.0)	132(12.0)	19(1.7)	

**Notes.**

Values were shown as the number (proportions) for categorical data. *P-value was calculated by Fisher’s exact test.

### Preliminary analysis

After testing normal distribution, perceived life stress (Skewness coefficient = −0.146, kurtosis coefficient = −0.163), depressive symptoms (Skewness coefficient = 0.321, kurtosis coefficient = −0.687) and subjective well-being (Skewness coefficient = −0.062, kurtosis coefficient = −0.230) was found to obey approximately a normal distribution. Table 2 shows the linear regression analysis results of the relationships of perceived life stress, depressive symptoms and interests/hobbies with subjective well-being. After adjusting confounding factors, it was found that perceived life stress (*B* =  − 1.424, *β* = −0.101, *P* < 0.001) and depressive symptoms (*B* =  − 0.569, *β* = −0.470, *P* < 0.001) were associated with subjective well-being. The mean scores of depressive symptoms and subjective well-being were 43.89 (SD = 10.61) and 89.94 (SD = 12.84), respectively. Perceived life stress (*r* =  − 0.289, *P* < 0.001) and depressive symptoms (*r* =  − 0.606, *P* < 0.001) were negatively associated with subjective well-being. Perceived life stress was positively associated with depressive symptoms (*r* = 0.246, *P* < 0.001). Data are shown in Table 3.

**Table 2:** Linear regression analysis of the perceived life stress, depressive symptoms, and interests/hobbies with subjective well-being.

**Variable**	***B***	***SE***	*β*	***t***	***R*** ^2^	***F***
**Constant**	111.930	2.857	–	39.174^***^	0.431	51.446^***^
**Perceived life stress**	−1.424	0.361	−0.101	−3.949^***^		
**Depressive symptoms**	−0.569	0.035	−0.470	−16.181^***^		
**Interests/hobbies**	2.719	0.695	0.094	3.910^***^		

**Notes.**

The results adjusted for age, marital status, employment status, educational level, average monthly personal income, self-rated health, smoking status, drinking status, self-rated good family relationship, menstrual status, the use of progesterone/estrogen therapy, sleep quality and anxiety symptoms.

*** *P* < 0.001.

**Table 3:** Descriptive and correlations between perceived life stress, depressive symptoms and subjective well-being.

**Variables**	**M**	**SD**	**1**	**2**	**3**
**1. Perceived life stress**	–	–	1		
**2. Depressive symptoms**	43.89	10.61	0.246^***^	1	
**3. Subjective well-being**	89.94	12.84	−0.289^***^	−0.606^***^	1

**Notes.**

*** *P* < 0.001.

### Testing for mediation effect

A multiple linear regression analysis was employed to explore whether depressive symptoms mediate the association between perceived life stress and subjective well-being for testing the mediation effects. The confounding factors were adjusted in each mediation effect analysis. Table 4 displays the results of multiple linear regressions. Firstly, perceived life stress was positively related to depressive symptoms (*β* = 0.110, *P* < 0.001) in Model 1. Secondly, perceived life stress was negatively associated with subjective well-being (*β* = −0.154, *P* < 0.001) in Model 2. Thirdly, depressive symptoms had a negative predictive effect on subjective well-being (*β* = −0.487, *P* < 0.001), controlling for the direct impact of perceived life stress on subjective well-being in Model 3. Although remaining significant, the effect of perceived life stress on subjective well-being was reduced (*β* = −0.100, *P* < 0.001).

**Table 4:** Mediated regression analysis for perceived life stress and depressive symptoms as predictors of subjective well-being.

**Variable**	**Model 1:** **Depressive symptoms**			**Model 2:** **Subjective well-being**			**Model 3:** **Subjective well-being**		
	B	*β*	*t*	B	*β*	*t*	B	*β*	*t*
**Perceived life stress**	1.290	0.110	4.133^***^	−2.173	−0.154	−5.376^***^	−1.412	−0.100	−3.892^***^
**Depressive symptoms**							−0.590	−0.487	−16.870^***^
***R*** ^2^	0.364			0.272			0.423		
**F**	44.609^***^			29.060^***^			53.158^***^		

**Notes.**

The results adjusted for age, marital status, employment status, educational level, average monthly personal income, self-rated health, smoking status, drinking status, self-rated good family relationship, menstrual status, the use of progesterone/estrogen therapy, sleep quality and anxiety symptoms.

*** *P* < 0.001.

Simple mediation analysis (Model 4) reconfirmed the relationships of perceived life stress and depressive symptoms with subjective well-being. The indirect effects of perceived life stress on subjective well-being through depressive symptoms (Effect = −0.760, 95% CI [−1.129 to −0.415]) were significant, as zero was not contained in the 95% CI. Hence, we confirmed that depressive symptoms partial mediates the association between perceived life stress and subjective well-being.

### Testing for moderated mediation effects

A moderated mediation model was established based on the mediation model. The confounding factors were adjusted in each analysis. Firstly, Model 1 was significant (*F* = 48.544, *P* < 0.001, *R*^2^ = 0.432). Perceived life stress was negatively associated with subjective well-being, whereas interests/hobbies were positively associated with subjective well-being. The interaction between perceived life stress and interests/hobbies was unrelated to subjective well-being, suggesting that interests/hobbies failed to moderate the association between perceived life stress and subjective well-being. Secondly, Model 2 was significant (*F* = 41.530, *P* < 0.001, *R*^2^ = 0.379). Perceived life stress was positively associated with depressive symptoms, whereas interests/hobbies were negatively associated with depressive symptoms. The interaction between perceived life stress and interests/hobbies was unrelated to depressive symptoms, indicating that interests/hobbies failed to moderate the association between perceived life stress and depressive symptoms. Finally, Model 3 was significant (*F* = 49.027, *P* < 0.001, *R*^2^ = 0.434). Perceived life stress (*β* = −0.099, *P* < 0.001) and depressive symptoms (*β* = −0.473, *P* < 0.001) were negatively related to subjective well-being, whereas interests/hobbies ware positively associated with subjective well-being (β = 0.077, *P* < 0.01). The impact of the interaction term between depressive symptoms and interests/hobbies on subjective well-being showed statistical significance (*β* = −0.060, *P* < 0.05), thereby indicating the moderating effect of interests/hobbies on the relationship between depressive symptoms and subjective well-being. Data are shown in Table 5.

**Table 5:** Testing the moderated mediation analysis results.

**Variable**	**Model 1:** **Subjective well-being**			**Model 2**: **Depressive symptoms**			**Model 3:** **Subjective well-being**		
	B	*β*	*t*	B	*β*	*t*	B	*β*	*t*
**Perceived life stress**	−1.421	−0.100	−3.942^***^	1.272	0.109	4.120^***^	−1.401	−0.099	−3.895^***^
**Interests/hobbies**	2.775	0.096	3.983^***^	−3.035	−0.127	−5.107^***^	2.239	0.077	3.111^**^
**Perceived life stress** ×**Interests/hobbies**	0.943	0.029	1.279	−0.080	−0.003	−0.126			
**Depressive symptoms**	−0.569	−0.470	−16.181^***^				−0.572	−0.473	−16.301^***^
**Depressive symptoms** ×**Interests/hobbies**							−0.171	−0.060	−2.511^*^
***R*** ^2^	0.432			0.379			0.434		
**F**	48.544^***^			41.530^***^			49.027^***^		

**Notes.**

The results adjusted for age, marital status, employment status, educational level, average monthly personal income, self-rated health, smoking status, drinking status, self-rated good family relationship, menstrual status, the use of progesterone/estrogen therapy, sleep quality, anxiety symptoms.

* *P* < 0.05.

** *P* < 0.01.

*** *P* < 0.001.

**Figure 2:**
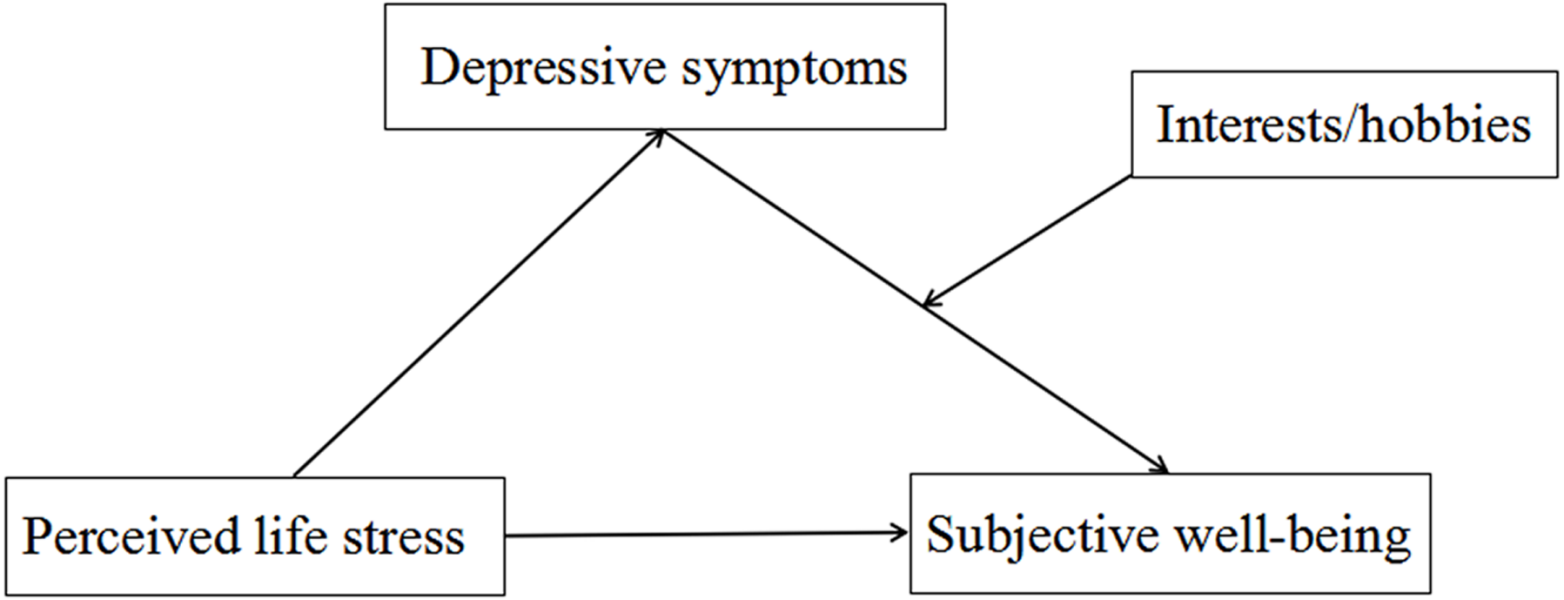
The framework of the moderated mediation model in this study.

In order to examine this stage of moderated mediation, PROCESS Model 14 was used for reconfirming the result. Moderated mediation had a significant index (Index = −0.220, SE = 0.099, 95% CI [−0.460 to −0.060]), implying that the indirect effect of perceived life stress on subjective well-being was moderated by interests/hobbies. More specifically, interests/hobbies moderated the association between depressive symptoms and subjective well-being. The conditional indirect effect of perceived life stress on subjective well-being was significant for both groups with and without extensive interests/hobbies group (Effect = −0.899, SE = 0.223, 95% CI [−1.349 to −0.475], and Effect = −0.679, SE = 0.169, 95% CI [−1.027 to −0.363]). Figure 2 shows the framework of the moderated mediation model in this study.

**Figure 3:**
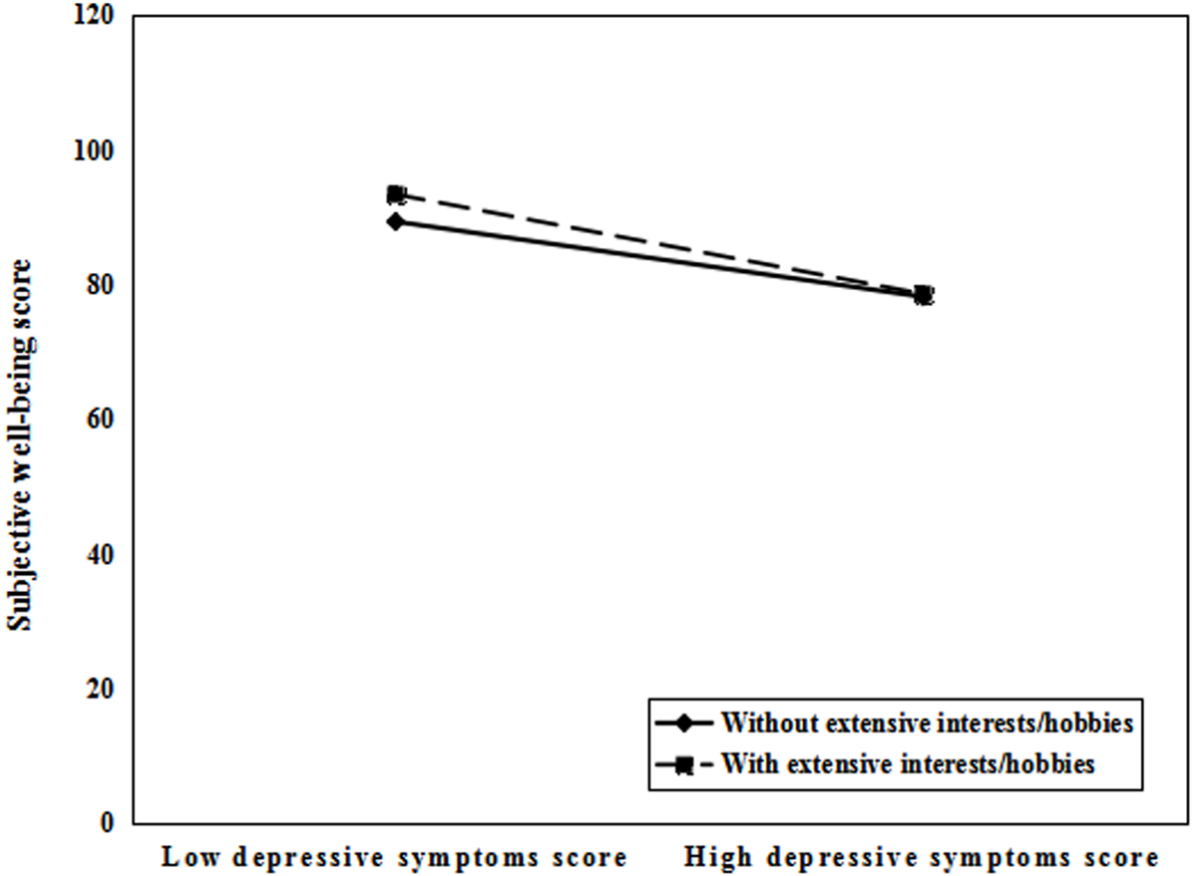
Interests/hobbies moderated the effect of depressive symptoms on subjective well-being.

As displayed in Fig. 3, the simple slope tests demonstrated that, for perimenopausal women without extensive interests/hobbies, the effect of depressive symptoms and subjective well-being was significant (B_simple_ = −0.572, *t* =  − 16.301, *P* < 0.001). However, for individuals with extensive interests/hobbies, the effect of depressive symptoms and subjective well-being was still significant but considerably stronger (B_simple_ = −0.743, *t* =  − 9.565, *P* < 0.001).

## Discussion

There were three major findings in this study. First, perceived life stress was negatively associated with subjective well-being. Second, depressive symptoms partially mediated the association between perceived life stress and subjective well-being. Third, interests/hobbies moderated the indirect effect of depressive symptoms on the association of perceived life stress with subjective well-being. These findings provide valuable information to promote subjective well-being among perimenopausal women and help to formulate the targeted interventions for individuals and health policy departments.

As expected, perceived life stress has a negative association with subjective well-being. This suggested that as the level of perceived life stress increases, the subjective well-being in perimenopausal women becomes worse, which verified Hypothesis 1. A longitudinal analysis suggested that well-being is negatively affected by stress during the menopausal transition (Dennerstein et al., 2007). High levels of perceived life stress could lead to difficulties of perimenopausal women in interpersonal communication and are associated with lower life satisfaction, which would decrease subjective well-being. In this sense, it would be very positive for perimenopausal women to develop stress management abilities to increase subjective well-being.

The mediating analysis indicated that depressive symptoms partially mediated the association between perceived life stress and subjective well-being, which confirmed Hypothesis 2. Perimenopausal women with a high level of perceived life stress were more likely to experience depressive symptoms, which in turn resulted in a decrease in subjective well-being. To be specific, perceived life stress was positively associated with depressive symptoms among perimenopausal women. Not surprisingly, as daily stress increased, individuals were more likely to report higher negative and lower positive affects (Richardson, 2017). A similar study found that high-level perceived stress was related to depressive symptoms (Mitchell & Woods, 2017). Models of the stress-depression relationship highlighted the importance of how stressors were perceived cognitively (Dulaney et al., 2018). As posited by cognitive theories of depression, people’s inferences, thoughts, attitudes, interpretations and ways of attending to and recalling events can increase their development and recurrence risks of depressive episodes (Gotlib & Joormann, 2010). Perimenopausal women with high perceived life stress are likely to adopt more negative coping styles and produce more passive attitudes or thoughts when dealing with problems, thereby increasing the probability of depressive symptoms. The presence of depressive symptoms was a negative influencing factor for subjective well-being. Depressive symptoms had been proved to be associated with functional limitation (Hairi et al., 2011), mortality (Correa et al., 2021), and cardiovascular disease (Haigh et al., 2020). In addition, because of generating more negative emotions and relating to avoidant coping (Thimm et al., 2018), depressive symptoms exert a negative impact on subjective well-being. Therefore, higher perceived life stress might reduce the subjective well-being among perimenopausal women by increasing depressive symptoms.

More importantly, the results found that interests/hobbies moderated the indirect effect of depressive symptoms on the relationship between perceived life stress and subjective well-being. Specifically, interests/hobbies buffered the negative impact of depressive symptoms on subjective well-being, which proved the broaden-and-build theory of positive emotions (Fredrickson, 2004). Larger indirect effects were observed among perimenopausal women with extensive interests/hobbies than those without extensive interests/hobbies. Perimenopausal women with extensive interests/hobbies can strengthen social and psychological resources and improve problem-solving capabilities, which thus can offset and regulate the negative effects of depressive symptoms on subjective well-being. As a result, it is advised to train and encourage extensive and diversified interests/hobbies to alleviate the negative effects of depressive symptoms and promote the improvement of subjective well-being.

Several limitations should be noted. First, this study was based on a cross-sectional design, which cannot provide strong evidence for causality. Second, it should be noted that all the data stemmed from the self-reported questionnaires of the respondents, which may exist the problems of subjectivity and recall bias.

These findings have several practical implications. Health care providers should recognize the importance of perceived life stress and depressive symptoms to improve the subjective well-being of perimenopausal women. It is suggested to adopt a wide range of strategies like psychological counseling, enhanced coping ability of individuals with stress in daily life, and early mental health interventions to relieve or prevent negative emotions, ultimately promoting subjective well-being of perimenopausal women. Furthermore, the broaden-and-build theory of positive emotions emphasizes the importance of positive emotions for undoing negative emotions and promoting psychological well-being. Perimenopausal women should cultivate a broad range of interests/hobbies in their own lives to develop experiences of positive emotions, which is also conducive to improving subjective well-being.

## Conclusions

Perceived life stress was negatively associated with subjective well-being. The impact of perceived life stress on subjective well-being was partially mediated by depressive symptoms, and interests/hobbies moderated the indirect effect of depressive symptoms on the association between perceived life stress and subjective well-being. In this study, the findings may expand people’s knowledge on the association between perceived life stress and subjective well-being among perimenopausal women, and form an effective way to improve subjective well-being.

## Supplemental Information

10.7717/peerj.12787/supp-1 Supplemental Information 1 Data, code and questionnaire

## Additional Information and Declarations

### Data Availability

The following information was supplied regarding data availability: The raw measurements are available in the Supplementary File.
