# Supplementary material for: Association between perceived life stress and subjective well-being among Chinese perimenopausal women: a moderated mediation analysis
Source: PeerJ. 2022 Jan 18;10:e12787. doi: 10.7717/peerj.12787 (PMC8781442; doi:10.7717/peerj.12787)
Supplement: Supplemental Information 1 [file peerj-10-12787-s001.zip › supplemental_file_1/supplemental file 1/Code.docx]

**The codebook that converts numbers to their respective factors is as follows:**

| Variables | Code | Code | Code | Code |
| --- | --- | --- | --- | --- |
| Age | 1=“40-44” | 2=“45-50” | 3=“51-55” | 4=“56-60” |
| Marital status | 0=“Unmarried” | 1=“Married” |  |  |
| Employment status | 0=“Unemployed” | 1=“Employed” |  |  |
| Educational level | 1=“High school and below” | 2=“Junior college” | 3=“Bachelor degree and above” |  |
| Average monthly personal income | 1=“＜3,999” | 2=“4,000-7,999” | 3=“≥8,000” |  |
| Self-rated health | 0=“Poor” | 1= “Fair” | 2= “Good” |  |
| Smoking status | 0=“No” | 1=“Yes” |  |  |
| Drinking status | 0=“No” | 1=“Yes” |  |  |
| Menstrual status | 1=“Regular menstruation” | 2=“Irregular menstruation” | 3=“Menopause” |  |
| Progesterone/estrogen therapy | 0=“No” | 1=“Yes” |  |  |
| Sleep quality | 0=“Good” | 1=“Poor” |  |  |
| Anxiety symptoms | 0=“No” | 1=“Yes” |  |  |
| Self-rated good family relationship | 0=“No” | 1=“Yes” |  |  |
| Interests/hobbies | 0=“No” | 1=“Yes” |  |  |
| Perceived life stress(continuous) |  |  |  |  |
| Depression symptoms (continuous) |  |  |  |  |
| Subjective well-being (continuous) |  |  |  |  |
