# Supplementary material for: Association between perceived life stress and subjective well-being among Chinese perimenopausal women: a moderated mediation analysis
Source: PeerJ. 2022 Jan 18;10:e12787. doi: 10.7717/peerj.12787 (PMC8781442; doi:10.7717/peerj.12787)
Supplement: Supplemental Information 1 [file peerj-10-12787-s001.zip › supplemental_file_1/supplemental file 1/Questionnaire_(in_English).docx]

**Perimenopausal women** **health questionnaire**

**Personal information（A）**

A1. Your date of birth (Gregorian calendar): Year Month Date

A2. Your marital status:

① Unmarried ② Cohabitation/Married (including remarriage)

③ Divorce/Separation ④ Widowed

A3. Your educational level:

① Junior high school and below

② High school/technical secondary school/vocational high school

③ Junior college ④ Bachelor degree ⑤ Postgraduate and above

A4. Your professional status:

① Employed ② Retired ③ Unemployed

A5. Average monthly personal income:

① ≤1999 RMB ② 2000-3999 RMB ③ 4000-5999 RMB

④ 6000-7999 RMB ⑤ 8000-9999 RMB ⑥ ≥10000 RMB

**Behavior and lifestyle (B)**

B1. Your smoking situation:

① Smoking now ② Smoking past ③ Never smoking

B2. Your drinking situation:

① Drinking now ② Drinking past ③ Never drinking

**Self-rated health (C)**

C1. How do you rate your current health?

① Very good ② Good ③ Fair ④ Poor ⑤ Very poor

C2. How do you rate your current life stress?

① Very low ② Low ③ Fair ④ High ⑤ Very high

C3. How do you rate your interests/hobbies?

① Very extensive ② Extensive ③ Fair

④ Not extensive ⑤ Very not extensive

C4. How do you rate your family relationship?

① Very poor ② Poor ③ Fair ④ Good ⑤ Very good

**Female health status (D)**

D1. Your current menstrual status: (Reminder: menstrual cycle refers to the time between two menstrual periods, and menstrual length refers to the time from the beginning of bleeding to the end of a menstrual period)

① Regular menstruation (compared with the past, there is no obvious change in menstrual cycle, length and quantity)

② Irregular menstruation (the interval between two menstruations changes ≥ 7 days and occurs again within 10 cycles after the first occurrence)

③ Very irregular menstruation (compared to the past, one menopause occurred for ≥2 months)

④ Menopause (menstruation stops for at least 12 months)

D2. Have you used estrogen/progestogen therapy?

① Yes ② No

**Self-rating Anxiety scale (E)**

E1. I feel more nervous and anxious than usual.

① None or a little of the time ② Some of the time

③ Good part of the time ④ Most or all of the time

E2. I feel afraid for no reason at all.

① None or a little of the time ② Some of the time

③ Good part of the time ④ Most or all of the time

E3. I get upset easily or feel panicky.

① None or a little of the time ② Some of the time

③ Good part of the time ④ Most or all of the time

E4. I feel like I'm falling apart and going to pieces.

① None or a little of the time ② Some of the time

③ Good part of the time ④ Most or all of the time

E5. I feel that everything is all right and nothing bad will happen.

① None or a little of the time ② Some of the time

③ Good part of the time ④ Most or all of the time

E6. My arms and legs shake and tremble.

① None or a little of the time ② Some of the time

③ Good part of the time ④ Most or all of the time

E7. I am bothered by headaches, neck and back pains.

① None or a little of the time ② Some of the time

③ Good part of the time ④ Most or all of the time

E8. I feel weak and get tired easily.

① None or a little of the time ② Some of the time

③ Good part of the time ④ Most or all of the time

E9. I feel calm and can sit still easily.

① None or a little of the time ② Some of the time

③ Good part of the time ④ Most or all of the time

E10. I can feel my heart beating fast.

① None or a little of the time ② Some of the time

③ Good part of the time ④ Most or all of the time

E11. I am bothered by dizzy spells.

① None or a little of the time ② Some of the time

③ Good part of the time ④ Most or all of the time

E12. I have fainting spells or feel like it.

① None or a little of the time ② Some of the time

③ Good part of the time ④ Most or all of the time

E13. I can breathe in and out easily.

① None or a little of the time ② Some of the time

③ Good part of the time ④ Most or all of the time

E14. I get feelings of numbness and tingling in my fingers, toes.

① None or a little of the time ② Some of the time

③ Good part of the time ④ Most or all of the time

E15. I am bothered by stomachaches or indigestion.

① None or a little of the time ② Some of the time

③ Good part of the time ④ Most or all of the time

E16. I have to empty my bladder often.

① None or a little of the time ② Some of the time

③ Good part of the time ④ Most or all of the time

E17. My hands are usually dry and warm.

① None or a little of the time ② Some of the time

③ Good part of the time ④ Most or all of the time

E18. My face gets hot and blushes.

① None or a little of the time ② Some of the time

③ Good part of the time ④ Most or all of the time

E19. I fall asleep easily and get a good night's rest.

① None or a little of the time ② Some of the time

③ Good part of the time ④ Most or all of the time

E20. I have nightmares.

① None or a little of the time ② Some of the time

③ Good part of the time ④ Most or all of the time

**Pittsburgh sleep quality index (F)**

The following questions are about your sleep in the last month. Please select or fill in the answers that best meet your actual situation in the last month. Please answer the following questions:

F1. In the past one month, I usually go to bed at o'clock at night.

F2. In the past one month, it usually takes minutes from going to bed to falling asleep.

F3. In the past one month, I usually get up at o'clock in the morning.

F4. In the past one month, the actual sleep hours every night. (not equal to bed time).

For the following questions, please choose the one that suits you best.

F5. In the past one month, I have been troubled by the following conditions affecting sleep:

F5a. Difficulty falling asleep (not falling asleep within 30 minutes).

① None ② <1 times/week ③ 1-2 times/week ④ ≥3 times/week

F5b. Waking up easily or early at night.

① None ② <1 times/week ③ 1-2 times/week ④ ≥3 times/week

F5c. Go to the toilet at night.

① None ② <1 times/week ③ 1-2 times/week ④ ≥3 times/week

F5d. Dyspnea.

① None ② <1 times/week ③ 1-2 times/week ④ ≥3 times/week

P5e. Cough or snore loudly.

① None ② <1 times/week ③ 1-2 times/week ④ ≥3 times/week

F5f. Feeling cold.

① None ② <1 times/week ③ 1-2 times/week ④ ≥3 times/week

F5g. Feeling hot.

① None ② <1 times/week ③ 1-2 times/week ④ ≥3 times/week

F5h. Have a nightmare.

① None ② <1 times/week ③ 1-2 times/week ④ ≥3 times/week

F5j. Pain and discomfort.

① None ② <1 times/week ③ 1-2 times/week ④ ≥3 times/week

F5k. Other things that affect sleep.

① None ② <1 times/week ③ 1-2 times/week ④ ≥3 times/week

If yes, please specify:

F6. Generally, what do you think of your sleep quality in the past one month?

① Very good ② Good ③ Poor ④ Very poor

F7. In the past one month, how did you use drugs for hypnosis?

① None ② <1 times/week ③ 1-2 times/week ④ ≥3 times/week

F8. In the past one month, have you often felt sleepy?

① None ② <1 times/week ③ 1-2 times/week ④ ≥3 times/week

F9. In the past one month, have you not had enough energy to do things?

① No ② Occasionally ③ Sometimes ④ Always

**Self-Rating Depression Scale (G)**

G1. I feel down-hearted and blue.

① A little of the time ② Some of the time

③ Good part of the time ④ Most of the time

G2. Morning is when l fell the best.

① A little of the time ② Some of the time

③ Good part of the time ④ Most of the time

G3. I have crying spells or feel like it.

① A little of the time ② Some of the time

③ Good part of the time ④ Most of the time

G4. I have trouble sleeping at night.

① A little of the time ② Some of the time

③ Good part of the time ④ Most of the time

G5. I eat as much as I used to.

① A little of the time ② Some of the time

③ Good part of the time ④ Most of the time

G6. I still enjoy sex.

① A little of the time ② Some of the time

③ Good part of the time ④ Most of the time

G7. I notice that I am losing weight.

① A little of the time ② Some of the time

③ Good part of the time ④ Most of the time

G8. I have trouble with constipation.

① A little of the time ② Some of the time

③ Good part of the time ④ Most of the time

G9. My heart beats faster than usual.

① A little of the time ② Some of the time

③ Good part of the time ④ Most of the time

G10. I get tired for no reason.

① A little of the time ② Some of the time

③ Good part of the time ④ Most of the time

G11. My mind is as clear as it used to be.

① A little of the time ② Some of the time

③ Good part of the time ④ Most of the time

G12. I find it easy to do things I used to.

① A little of the time ② Some of the time

③ Good part of the time ④ Most of the time

G13. I am restless and can’t keep still.

① A little of the time ② Some of the time

③ Good part of the time ④ Most of the time

G14. I fell hopeful about the future.

① A little of the time ② Some of the time

③ Good part of the time ④ Most of the time

G15. I am more irritable than usual.

① A little of the time ② Some of the time

③ Good part of the time ④ Most of the time

G16. I find it easy to make decisions.

① A little of the time ② Some of the time

③ Good part of the time ④ Most of the time

G17. I fell that I am useful and needed.

① A little of the time ② Some of the time

③ Good part of the time ④ Most of the time

G18. My life is pretty full.

① A little of the time ② Some of the time

③ Good part of the time ④ Most of the time

G19. I fell that others would be better off if I were dead.

① A little of the time ② Some of the time

③ Good part of the time ④ Most of the time

G20. I still enjoy the things I used to do.

① A little of the time ② Some of the time

③ Good part of the time ④ Most of the time

**Subjective Well-being Scale for Chinese Citizens (H)**

H1. The society will provide more and more outlets for people.

① Strongly disagree ② Disagree ③ A little disagree

④ A little agree ⑤ Agree ⑥ Strongly agree

H2. As age older, I have learned a lot of truths from life, which make I become stronger and more capable.

① Strongly disagree ② Disagree ③ A little disagree

④ A little agree ⑤ Agree ⑥ Strongly agree

H3. Most of the life goals I set can encourage me, not discourage.

① Strongly disagree ② Disagree ③ A little disagree

④ A little agree ⑤ Agree ⑥ Strongly agree

H4. I often feel like I am just fooling around.

① Strongly disagree ② Disagree ③ A little disagree

④ A little agree ⑤ Agree ⑥ Strongly agree

H5. I don't know the meaning of what I have done in my life.

① Strongly disagree ② Disagree ③ A little disagree

④ A little agree ⑤ Agree ⑥ Strongly agree

H6. I often feel that some parts of my body are particularly uncomfortable.

① Strongly disagree ② Disagree ③ A little disagree

④ A little agree ⑤ Agree ⑥ Strongly agree

H7. Compared with the people around me, I am content.

① Strongly disagree ② Disagree ③ A little disagree

④ A little agree ⑤ Agree ⑥ Strongly agree

H8. I am satisfied with my family's income.

① Strongly disagree ② Disagree ③ A little disagree

④ A little agree ⑤ Agree ⑥ Strongly agree

H9. I often worry about small things.

① Strongly disagree ② Disagree ③ A little disagree

④ A little agree ⑤ Agree ⑥ Strongly agree

H10. I am very upset about my health.

① Strongly disagree ② Disagree ③ A little disagree

④ A little agree ⑤ Agree ⑥ Strongly agree

H11. I often find it difficult to establish friendship with others.

① Strongly disagree ② Disagree ③ A little disagree

④ A little agree ⑤ Agree ⑥ Strongly agree

H12. I prefer my own personality.

① Strongly disagree ② Disagree ③ A little disagree

④ A little agree ⑤ Agree ⑥ Strongly agree

H13. I feel as if most people are more than my friends.

① Strongly disagree ② Disagree ③ A little disagree

④ A little agree ⑤ Agree ⑥ Strongly agree

H14. I feel very happy with my family.

① Strongly disagree ② Disagree ③ A little disagree

④ A little agree ⑤ Agree ⑥ Strongly agree

H15. My luck is worse than others.

① Strongly disagree ② Disagree ③ A little disagree

④ A little agree ⑤ Agree ⑥ Strongly agree

H16. I feel confident in the development of society.

① Strongly disagree ② Disagree ③ A little disagree

④ A little agree ⑤ Agree ⑥ Strongly agree

H17. Compared with the people around me, I feel that I suffer a lot.

① Strongly disagree ② Disagree ③ A little disagree

④ A little agree ⑤ Agree ⑥ Strongly agree

H18. When encountering unhappy things, I can't keep my spirits up for a long time.

① Strongly disagree ② Disagree ③ A little disagree

④ A little agree ⑤ Agree ⑥ Strongly agree

H19. I am pleased that my views have become more and more mature over the years.

① Strongly disagree ② Disagree ③ A little disagree

④ A little agree ⑤ Agree ⑥ Strongly agree

H20. Sometimes I find it difficult to communicate with my family (including parents, lovers, children, etc.).

① Strongly disagree ② Disagree ③ A little disagree

④ A little agree ⑤ Agree ⑥ Strongly agree
