# Supplementary material for: Association between perceived life stress and subjective well-being among Chinese perimenopausal women: a moderated mediation analysis
Source: PeerJ. 2022 Jan 18;10:e12787. doi: 10.7717/peerj.12787 (PMC8781442; doi:10.7717/peerj.12787)
Supplement: Supplemental Information 1 [file peerj-10-12787-s001.zip › supplemental_file_1/supplemental file 1/Questionnaire_(in_original_language).docx]

**围绝经期女性健康信息采集表**

**个人基本情况（A）**

A1. 您的出生年月（公历） 年 月 日

A2. 您的婚姻状况：

① 未婚 ② 同居/已婚(含再婚) ③ 离婚/分居 ④ 丧偶

A3. 您的文化程度：

① 初中及以下 ② 高中/中专/职高 ③ 大专

④ 本科 ⑤ 研究生及以上

A4. 您的职业状态：① 在职 ② 下岗 ③ 退休

A5. 家庭人均月收入：

① ≤1999元 ② 2000-3999元 ③ 4000-5999元

④ 6000-7999元 ⑤ 8000-9999元 ⑥ ≥10000元

**行为及生活方式（B）**

B1. 您的吸烟情况：

① 现在吸 ② 既往吸 ③ 从不吸

B2. 您的饮酒情况：

① 现在饮酒 ② 既往饮酒 ③ 不饮酒

**自评健康状况（C）**

C1. 您对目前的健康状况的自我评价：

① 非常好 ② 较好 ③ 一般 ④ 较差 ⑤ 非常差

C2. 您对目前的生活压力情况的自我评价：

① 非常小 ② 较小 ③ 一般 ④ 较大 ⑤ 非常大

C3. 您的兴趣和爱好:

① 非常广泛 ② 较广泛 ③ 一般 ④ 较不广泛 ⑤ 非常不广泛

C4. 您对目前家庭关系的自我评价是：

① 非常好 ② 较好 ③ 一般 ④ 较差 ⑤ 非常差

**女性健康情况（D）**

D1. 您目前的月经状态：（提示：月经周期指两次月经间隔的时间，月经长度指一次月经从开始出血到结束的时间）

①月经规律（与既往相比，月经周期、长短、量均无明显改变） (跳至G7)

②月经不规律（两次月经间隔时间改变≥7天，且首次出现后的10个周期内再次发生）(跳至G7)

③月经非常不规律（与既往相比，发生过一次停经时间≥2个月）(跳至G7)

④绝经（月经停止至少12个月）

D2. 您是否使用了雌/孕激素治疗：①是 ②否

**焦虑自评量表（E）**

E1. 我觉得比平常容易紧张或着急

①没有或很少 ②有时 ③大部分时间 ④绝大部分时间

E2. 我无缘无故地感到害怕

①没有或很少 ②有时 ③大部分时间 ④绝大部分时间

E3. 我容易心里烦乱或觉得惊恐

①没有或很少 ②有时 ③大部分时间 ④绝大部分时间

E4. 我觉得我可能将要发疯

①没有或很少 ②有时 ③大部分时间 ④绝大部分时间

E5. 我觉得一切都好，也不会发生什么不幸

①没有或很少 ②有时 ③大部分时间 ④绝大部分时间

E6. 我手脚发抖打颤

①没有或很少 ②有时 ③大部分时间 ④绝大部分时间

E7. 我因为头痛、颈痛和背痛而苦恼

①没有或很少 ②有时 ③大部分时间 ④绝大部分时间

E8. 我感觉容易衰弱和疲乏

①没有或很少 ②有时 ③大部分时间 ④绝大部分时间

E9. 我觉得心平气和，并且容易安静坐着

①没有或很少 ②有时 ③大部分时间 ④绝大部分时间

E10. 我觉得心跳很快

①没有或很少 ②有时 ③大部分时间 ④绝大部分时间

E11. 我因为一阵阵头晕而苦恼

①没有或很少 ②有时 ③大部分时间 ④绝大部分时间

E12. 我有晕倒发作或觉得要晕倒似的

①没有或很少 ②有时 ③大部分时间 ④绝大部分时间

E13. 我呼气吸气都感到很容易

①没有或很少 ②有时 ③大部分时间 ④绝大部分时间

E14. 我的手脚麻木和刺痛

①没有或很少 ②有时 ③大部分时间 ④绝大部分时间

E15. 我因为胃痛和消化不良而苦恼

①没有或很少 ②有时 ③大部分时间 ④绝大部分时间

E16. 我常常要小便

①没有或很少 ②有时 ③大部分时间 ④绝大部分时间

E17. 我的手常常是干燥温暖的

①没有或很少 ②有时 ③大部分时间 ④绝大部分时间

E18. 我脸红发热

①没有或很少 ②有时 ③大部分时间 ④绝大部分时间

E19. 我容易入睡并且一夜睡得很好

①没有或很少 ②有时 ③大部分时间 ④绝大部分时间

E20. 我做恶梦

①没有或很少 ②有时 ③大部分时间 ④绝大部分时间

**匹兹堡睡眠质量量表（F）**

下列问题是关于您最近1个月的睡眠情况，请选择或填写最符合您近1个月实际情况的答案。请回答下列问题：

F1. 近1个月，晚上上床睡觉通常 点钟。

F2. 近1个月，从上床到入睡通常需要 分钟。

F3. 近1个月，通常早上 点起床。

F4. 近1个月，每夜通常实际睡眠 小时(不等于卧床时间)。

对下列问题请选择1个最适合您的答案。

F5. 近1个月，因下列情况影响睡眠而烦恼：

F5a. 入睡困难(30分钟内不能入睡)

①无 ②<1次/周 ③1-2次/周 ④≥3次/周

F5b. 夜间易醒或早醒

①无 ②<1次/周 ③1-2次/周 ④≥3次/周

F5c. 夜间去厕所

①无 ②<1次/周 ③1-2次/周 ④≥3次/周

F5d. 呼吸不畅

①无 ②<1次/周 ③1-2次/周 ④≥3次/周

F5e. 咳嗽或鼾声高

①无 ②<1次/周 ③1-2次/周 ④≥3次/周

F5f. 感觉冷

①无 ②<1次/周 ③1-2次/周 ④≥3次/周

F5g. 感觉热

①无 ②<1次/周 ③1-2次/周 ④≥3次/周

F5h. 做恶梦

①无 ②<1次/周 ③1-2次/周 ④≥ 3次/周

F5j. 疼痛不适

①无 ②<1次/周 ③1-2次/周 ④≥3次/周

F5k. 其它影响睡眠的事情

①无 ②<1次/周 ③1-2次/周 ④≥3次/周

如有，请说明：

F6. 近1个月，总的来说，您认为自己的睡眠质量

①很好 ②较好 ③较差 ④很差

F7. 近1个月，您用药物催眠的情况

①无 ②<1次/周 ③1-2次/周 ④≥3次/周

F8. 近1个月，您常感到困倦吗？

①无 ②<1次/周 ③1-2次/周 ④≥3次/周

F9. 近1个月，您做事情的精力不足吗？

①没有 ②偶尔有 ③有时有 ④经常有

**抑郁自评量表（G）**

G1. 我觉得闷闷不乐，情绪低沉

① 很少有 ② 有时有 ③ 大部分时间有 ④ 绝大部分时间有

G2. 我觉得一天之中早晨心情最好

① 很少有 ② 有时有 ③ 大部分时间有 ④ 绝大部分时间有

G3. 我想哭或者一阵阵的哭出来

① 很少有 ② 有时有 ③ 大部分时间有 ④ 绝大部分时间有

G4. 我晚上睡眠不好

① 很少有 ② 有时有 ③ 大部分时间有 ④ 绝大部分时间有

G5. 我吃得跟平常一样多

① 很少有 ② 有时有 ③ 大部分时间有 ④ 绝大部分时间有

G6. 我与异性亲密接触时和以往一样感到愉快

① 很少有 ② 有时有 ③ 大部分时间有 ④ 绝大部分时间有

G7. 我发觉我的体重在下降

① 很少有 ② 有时有 ③ 大部分时间有 ④ 绝大部分时间有

G8. 我有便秘的苦恼

① 很少有 ② 有时有 ③ 大部分时间有 ④ 绝大部分时间有

G9. 我心跳比平时快

① 很少有 ② 有时有 ③ 大部分时间有 ④ 绝大部分时间有

G10. 我无缘无故感到疲乏

① 很少有 ② 有时有 ③ 大部分时间有 ④ 绝大部分时间有

G11. 我的头脑跟平常一样清楚

① 很少有 ② 有时有 ③ 大部分时间有 ④ 绝大部分时间有

G12. 我觉得做以前经常做的事并没有困难

① 很少有 ② 有时有 ③ 大部分时间有 ④ 绝大部分时间有

G13. 我坐立不安，难以保持平静

① 很少有 ② 有时有 ③ 大部分时间有 ④ 绝大部分时间有

G14. 我对将来抱有希望

① 很少有 ② 有时有 ③ 大部分时间有 ④ 绝大部分时间有

G15. 我比平常容易激动

① 很少有 ② 有时有 ③ 大部分时间有 ④ 绝大部分时间有

G16. 我觉得做出决定是容易的

① 很少有 ② 有时有 ③ 大部分时间有 ④ 绝大部分时间有

G17. 我觉得自己是个有用的人，有人需要我

① 很少有 ② 有时有 ③ 大部分时间有 ④ 绝大部分时间有

G18. 我的生活过得很有意思

① 很少有 ② 有时有 ③ 大部分时间有 ④ 绝大部分时间有

G19. 我认为如果我死了别人会生活得好些

① 很少有 ② 有时有 ③ 大部分时间有 ④ 绝大部分时间有

G20. 平常感兴趣的事我仍然照样感兴趣

① 很少有 ② 有时有 ③ 大部分时间有 ④ 绝大部分时间有

**中国居民主观幸福感量表简本（H）**

H1. 社会给人们提供的出路会越来越多

① 很不同意 ② 不同意 ③ 有点不同意

④ 有点同意 ⑤ 同意 ⑥ 非常同意

H2. 随着年龄增长，我从生活中悟出了许多道理，这是我变得更坚强、更有能力

① 很不同意 ② 不同意 ③ 有点不同意

④ 有点同意 ⑤ 同意 ⑥ 非常同意

H3. 我设立的生活目标多数能够给我鼓劲，而不是泄气

① 很不同意 ② 不同意 ③ 有点不同意

④ 有点同意 ⑤ 同意 ⑥ 非常同意

H4. 我经常感觉到自己只是在混日子

① 很不同意 ② 不同意 ③ 有点不同意

④ 有点同意 ⑤ 同意 ⑥ 非常同意

H5. 我不清楚自己一生所做的事情有什么意义

① 很不同意 ② 不同意 ③ 有点不同意

④ 有点同意 ⑤ 同意 ⑥ 非常同意

H6. 我经常感到自己身体某些部位特别不舒服

① 很不同意 ② 不同意 ③ 有点不同意

④ 有点同意 ⑤ 同意 ⑥ 非常同意

H7. 与周围的人相比，我很知足

① 很不同意 ② 不同意 ③ 有点不同意

④ 有点同意 ⑤ 同意 ⑥ 非常同意

H8. 我对家里的收入感到满意

① 很不同意 ② 不同意 ③ 有点不同意

④ 有点同意 ⑤ 同意 ⑥ 非常同意

H9. 我常因一些小事而烦恼

① 很不同意 ② 不同意 ③ 有点不同意

④ 有点同意 ⑤ 同意 ⑥ 非常同意

H10. 我很为自己的健康状况感到苦恼

① 很不同意 ② 不同意 ③ 有点不同意

④ 有点同意 ⑤ 同意 ⑥ 非常同意

H11. 我常常感到自己很难与他人建立友谊

① 很不同意 ② 不同意 ③ 有点不同意

④ 有点同意 ⑤ 同意 ⑥ 非常同意

H12. 我比较喜欢自己的个性

① 很不同意 ② 不同意 ③ 有点不同意

④ 有点同意 ⑤ 同意 ⑥ 非常同意

H13. 我感到似乎大多数人都比我朋友多

① 很不同意 ② 不同意 ③ 有点不同意

④ 有点同意 ⑤ 同意 ⑥ 非常同意

H14. 和家人在一起，我感到特别愉快

① 很不同意 ② 不同意 ③ 有点不同意

④ 有点同意 ⑤ 同意 ⑥ 非常同意

H15. 我的运气比别人差

① 很不同意 ② 不同意 ③ 有点不同意

④ 有点同意 ⑤ 同意 ⑥ 非常同意

H16. 我对社会的发展感到很有信心

① 很不同意 ② 不同意 ③ 有点不同意

④ 有点同意 ⑤ 同意 ⑥ 非常同意

H17. 与周围人相比，我感到自己挺吃亏

① 很不同意 ② 不同意 ③ 有点不同意

④ 有点同意 ⑤ 同意 ⑥ 非常同意

H18. 碰到不开心的事情时，很长时间我都打不起精神来

① 很不同意 ② 不同意 ③ 有点不同意

④ 有点同意 ⑤ 同意 ⑥ 非常同意

H19. 我感到高兴的是，这些年自己的看法越来越成熟

① 很不同意 ② 不同意 ③ 有点不同意

④ 有点同意 ⑤ 同意 ⑥ 非常同意

H20. 我有时感到很难与家人（包括父母、爱人、孩子等）沟通

① 很不同意 ② 不同意 ③ 有点不同意

④ 有点同意 ⑤ 同意 ⑥ 非常同意
